# Risk perception and gratitude mediate the negative relationship between COVID-19 management satisfaction and public anxiety

**DOI:** 10.1038/s41598-023-29815-2

**Published:** 2023-02-27

**Authors:** Ying Mei, Lisha Tan, Wenmin Yang, Jie Luo, Lei Xu, Yi Lei, Hong Li

**Affiliations:** 1 Institution of Brain and Psychological Science, Sichuan Normal University, Chengdu, China; 2 Faculty of Education and Psychology, University of Jyväskylä, Jyväskylä, Finland; 3 Faculty of Psychology, Beijing Normal University, Beijing, China; 4 School of Psychology, Guizhou Normal University, Guiyang, China

**Keywords:** Health care, Psychology

## Abstract

In this study, we explored whether satisfaction with government management, perception of risk, and gratitude influenced public anxiety during the COVID-19 pandemic in China. Using a cross-sectional, anonymous and confidential online survey, a nationwide sample of Chinese adults (N = 876) was targeted between March 25–March 30, 2020, a period in which newly confirmed cases significantly declined in China. The anxiety level was decreased as compared to that assessed during the peak period. Multiple parallel mediation modeling demonstrated that risk perception and gratitude partially mediated the relationship between satisfaction with government management and public anxiety. Increasing satisfaction and gratitude, as well as reducing risk perception contribute to the public’s mental health. The results may shed light on the positive factors for psychological well-being during the COVID-19 pandemic and may aid potential strategies for the policy maker, the public, and the clinic to regulate negative emotions or future emerging infectious diseases.

## Introduction

The novel 2019 coronavirus disease (COVID-19) was first diagnosed in Wuhan and has caused a serious crisis in the world. Along with the considerable loss of lives and economic damage, the pandemic triggered severe public anxiety reactions (see Table 1). As shown by research, a large percentage of respondents reported anxiety symptoms associated with fear of infection, quarantine, and excessive exposure to related information, as well as concerns about loss of work, study, or income^1–3^. Public anxiety during the pandemic can lead to a high level of stress, impaired sleep quality^4^, harmful alcohol use^1^, and even self-harm or suicidal ideation^5^. Therefore, the need for understanding the possible predictive factors that reduce public anxiety is urgent.

**Table 1:** China’s general public anxiety during the initial and peak periods of the pandemic.

Author	Measure time	Frequency	Age	Tools	Result
Li et al., 2020	Feb 9–Feb 16	5033	Over 18 years old	GAD-7	The prevalence of anxiety was 20.4%, which was associated with COVID-19 related news
Lin et al., 2020	Jan 24–Feb 24	2446	18–70 years old	STAI	The majority of respondents showed high levels (a score of 40 or higher) of STAI-S (78.3%) and high levels of STAI-T (76.7%)
Huang & Zhao, 2020	Feb 3–Feb 17	7236	Average 35.3 ± 5.6 years	GAD-7	The average prevalence of anxiety was 35.1% among younger people reporting more symptoms
Ahmed et al., 2020	Before Mar 13	1074	14–68 years old	BAI	About 29% of respondents are suffering from different forms of anxiety (mild [10.1%], moderate [6.0%], and severe [12.9%]) related to mandatory quarantine
Wang et al., 2020	Jan 31–Feb 2; Feb 28–Mar 1	1738	12–59 years old	DASS-21	No significant difference between the first survey (6.16) and second survey (6.15) for the DASS-anxiety subscale was indicated
Shi et al., 2020	Feb 28–Mar 11	56,679	Over 18 years old	GAD-7	About 31.6% of participants reported anxiety symptoms, with 10.4% reporting moderate to severe anxiety. People who might have contact with COVID-19 patients or people with suspected infection are vulnerable groups
Ran et al., 2020	Feb 23–Mar 2	1770	Average 28.7 ± 10.64 years old	GAD-7	About 31.9% of respondents had anxiety symptoms, with 8.8% with moderate or severe symptoms
Zhao et al. 2020	Feb 2–Feb 6	2006	Over 13 years old	BAI	The high anxiety level was associated with quarantine, living in high epidemic areas, divorced/widowed, and work related to the medical system
Liu X et al., 2020	Jan 30–Feb 3	608	19–69 years old	STAI	The proportion of respondents reporting state anxiety (15.8%) was higher than that of trait anxiety (4.0%)
Chao et al., 2020	Jan 28	917	Average 28.6 ± 9.5 years old	DASS-21	The anxiety symptoms were associated with new media use

*GAD-7* Generalized Anxiety Disorder-7, *STAI* State-Trait Anxiety Inventory, *BAI* Beck Anxiety Inventory, *SCL-90* Symptom Checklist 90.

### Attitude toward government and public anxiety

The attitude toward the government plays an important role in effective prevention and mental health during the pandemic. Previous studies suggested that trust in government is positively related to compliance with protective policy and the intention to accept vaccination^6–8^, and has been considered as a primary factor shaping individual risk perception^9–11^. The more the people trusted the government, the less state anxiety they experienced during the 2003 severe acute respiratory syndrome (SARS) pandemic^12^. However, the pandemic itself not only relies on but may change trust in institutions^13–16^, which is so called ‘compensatory institutional trust’^17^. Therefore, considering trust in government as a predictor for public anxiety might be inappropriate.

A better alternative predictor is public satisfaction with government performance which reflects the subjective perceptions of what the government has done and prior expectations^18^. As the expectancy-disconfirmation model^19^ suggests, the public is satisfied when their perceptions of current performance exceed expectations and is dissatisfied when the performance falls short of their expectations. Previous studies indicated perception of and satisfaction with government performance could explain trust in government^18,20–22^. And one study has reported that satisfaction with the government is negatively related to negative emotionality and positively related to well-being in social workers during COVID-19^23^. Studies on life satisfaction also suggested satisfaction is a significant predictor of clinical anxiety^24^. Thus, the current study focused on how satisfaction with government control actions influences public anxiety during COVID-19.

### Mediating role of risk perception

Risk perception often refers to intuitive judgments people make to evaluate the probability that the crisis occurs and the severity of the damage^25^. In the context of disease pandemics, risk perception mediated government response and public compliance^26^. Findings also emphasize that risk perception is a significant predictor of mental health^27,28^. As for COVID-19, a higher perceived risk is likely to initiate and aggravate mental issues such as anxiety, stress, and depression^28–30^. Empirical evidence showed that great perceived risk of infection predicted higher level of anxiety among individuals^31,32^.

When knowledge and cognition are limited or the crisis is invisible, risk perception will be increased^33^. In the case of COVID-19, the situation was largely uncontrollable for individuals, and the attributes of the virus were completely unknown and invisible^34^. Therefore, individuals have to trust and follow the recommendations of scientists, medical institutions and government for risk management and behavioral adjustment initially^9^. Trust in government can reduce risk perception through decreasing the uncertainty caused by rare infectious diseases, and thereby reduce inappropriate public anxiety^9^. However, it can also lead to the opposite result if the trust is damaged^9^.

Satisfaction with government performance may impact risk perception since it predicts trust in government as mentioned previously^18,20,21^. A previous study showed that satisfaction with environmental governance was negatively associated with environmental risk perception^35^. One recent study also reported significant temporal changes in satisfaction with management entities, COVID-19 risk perception and anxiety^36^. Hence, we hypothesize that risk perception mediates the relationship between satisfaction with government performance and public anxiety.

### Mediating role of gratitude

Gratitude is a common positive emotion that can facilitate individuals’ restoration and growth after experiencing a traumatic event^37,38^. Wood and colleagues^39^ considered gratitude as a wider life orientation toward noticing and appreciating the positives in the world. It can stem from an appreciation of the simple aspects of life (such as waking up in the morning), or be activated when people receive positive outcomes from others^40,41^.

Research shows that gratitude is robustly associated with increased well-being and less depression and anxiety^39–42^. As for anxiety, the strength between gratitude and trait anxiety has reached moderate levels (*r* = [−0.28, −0.46])^43^. Furthermore, gratitude interventions had a medium effect on anxiety symptoms^42^, and have been applicated in reducing anxiety symptoms in the clinical sample, such as anxiety disorder and post-traumatic stress disorder^44–46^. The underlying mechanism might be that gratitude allows people to explain various stimuli and life events in positive terms instead of selectively focusing on the negative aspects of the self and the world^39^.

A greater sense of satisfaction may increase gratitude, and thereby reduce psychological problems. Satisfaction and gratitude mutually predict each other over time^47^. When people experience high levels of life satisfaction, they tend to evaluate things positively and are more likely to respond with gratitude^48^. Gratitude also increases when something goes beyond their social expectations^48^. It is possible that during the COVID-19 pandemic people may feel a strong sense of gratitude when outcomes of government control actions exceed their expectations. To date, few studies have examined the association between satisfaction with government performance and gratitude during public health events. Therefore, we hypothesized that gratitude would mediate the effect of satisfaction with government management on anxiety.

### Overview of the current study

**Figure 1:**
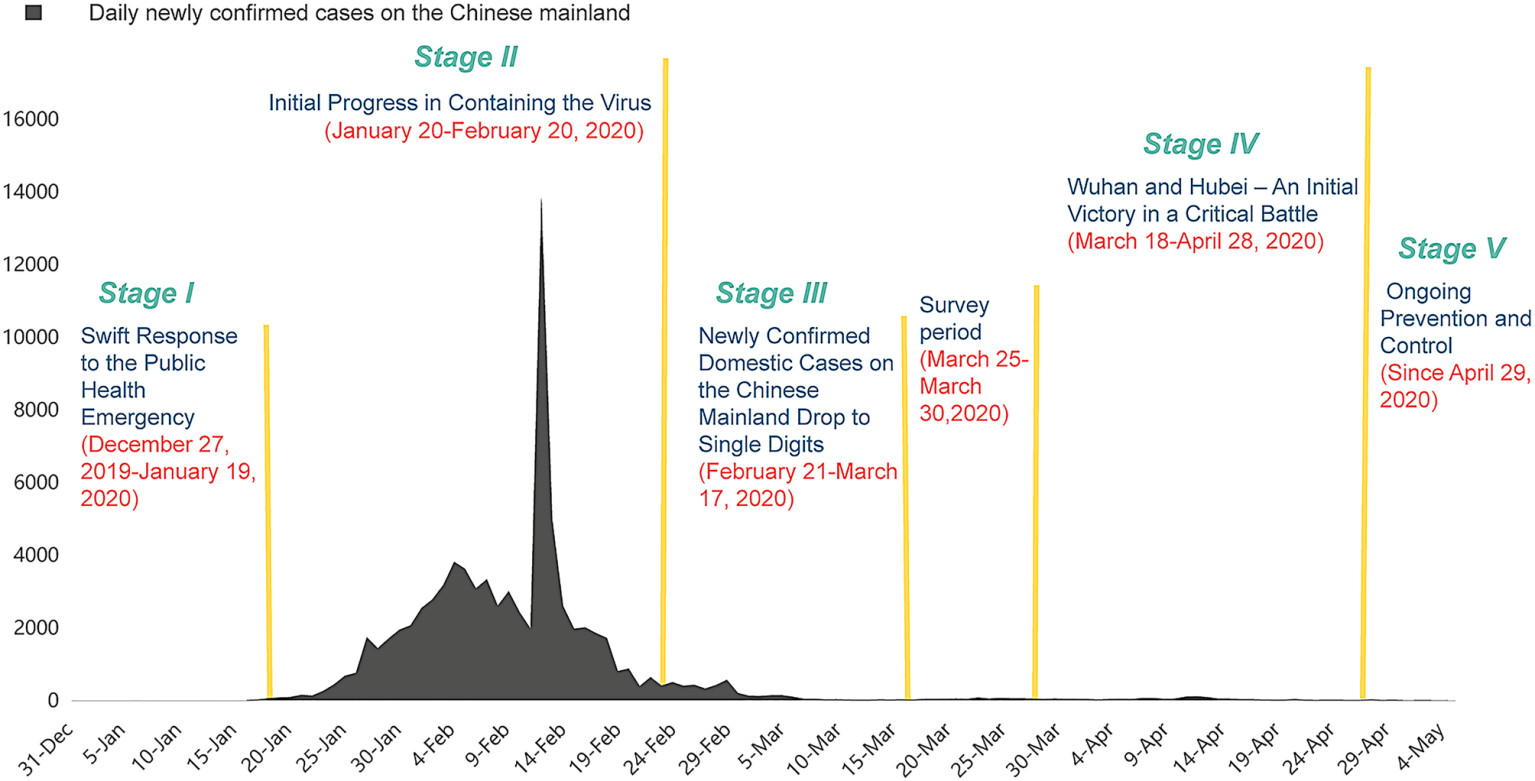
Daily figures for newly confirmed cases in Mainland China (from January 3 to May 5, 2020; Data sources: World Health Organization Situation Reports^49^) and stages of China’s fight against the epidemic (sources: Fighting Covid-19 China in Action^55^).

The data for the current study comes from a large-scale anonymous online survey conducted from March 25 to March 30, 2020. During this period, the spread of COVID-19 in China’s mainland slowed down and the entire country began to return to work. As shown in Fig. 1, the number of new confirmed cases declined substantially within four weeks in the entire country in March 2020^49,50^. No new cases have been reported for five consecutive days since March 19, in the epicenter, Wuhan, Hubei province^51–53^. And with the reopening of Hubei province on March 25, work and life gradually returned to normal^54^. The period of this survey was at the beginning of stage four of China’s fight against the epidemic^55^. The Chinese government has taken a series of measures to contain the infection, and the accomplishments proved effective and successful based on epidemiological data and empirical evidence, as noted above. Therefore, this period is a very good time to capture the changes in public attitude toward government management, risk perception, gratitude, as well as public anxiety.

The present study aimed to ascertain whether a positive attitude toward the government contributed to the alleviation of public anxiety during the pandemic. A multi-parallel mediation model is constructed in the present study to explore the effects of satisfaction with government management on public anxiety during the COVID-19 pandemic, particularly through risk perception and gratitude. We hypothesized that these two pandemic-related psychological variables would be significant mediators in the association between satisfaction with the government and public anxiety.

## Method

### Participants

This study obtained data from March 25 to 30, 2020 by using an anonymous online questionnaire through an online survey platform (https://www.wjx.cn; Changsha RanXing Science and Technology, Shanghai, China). Recruitment advertisements (in which we stated that the study was hosted by an academic institution rather than any government offices) were sent to participants through social media (“WeChat”, Tencent, Shenzhen, China), as well as the survey link. All participants were provided informed consent before their participation and compensated with 10 CNY after they completed the survey. To ensure participants respond honestly and seriously, they were informed at the very beginning that this survey is anonymous and no personally identifiable information will be collected (e.g. Name, ID, contacts, etc.), and that all their responses will be confidential and used for scientific research purposes only. Besides, two lie detection questions were included in the survey to filter unqualified data.

The current study was conducted in accordance with the Declaration of Helsinki. All material and procedures were reviewed and approved by the institutional review board of the Sichuan Normal University (protocol number: SCNU-20200301; can be acquired from the corresponding author upon reasonable request). A total of 935 random samples were drawn from the general public. In the end, 127 participants were excluded for being under 18 years old (*n* = 20) or answering too fast (less than two minutes; *n* = 39), or having unqualified/missing data (*n* = 68), leaving a total of 808 adult sample (315 males) for the statistical analysis. Information on their demographic characteristics (gender, age, current residency, educational attainment, coronavirus diagnosis, whether a relative was infected, and health information if under medical observation) were additionally acquired and reported in Table 2.

**Table 2:** Participants’ demographic characteristics (N = 808).

Variable	Attribute	Frequecy	Proportion (%)
Gender	Male	315	38.99
	Female	493	61.01
Age (years)	18–24	311	38.49
	25–30	110	13.61
	31–40	217	26.86
	41–50	110	13.61
	> 50	60	7.43
Current residence	Hubei Province	143	17.70
	Other regions	665	82.30
Education level	Junior high school and below	49	6.10
	High school/technical school	111	13.70
	College/university	461	57.10
	Postgraduate or above	187	23.10
Infected with coronavirus	Yes	1	0.12
	No	807	99.88
Medical observation period	Yes	8	0.99
	No	800	99.01
Relatives and friends infected	Yes	24	2.97
	No	784	97.03

### Measurements

#### Government Management Satisfaction Questionnaire

The self-developed Government Management Satisfaction Questionnaire (GMSQ) was used to measure the level of satisfaction with government control actions during the pandemic: “(1) The current pandemic would be awful without government actions; (2) I am very appreciative of what the state has done to prevent and control the pandemic; (3) I am proud of our current pandemic prevention and control achievements; (4) I think the pandemic is not managed primarily because the government’s prevention and controls are inadequate; (5) I support the government’s pandemic prevention and control actions; and (6) To prevent and control the pandemic, I am positively cooperating with the government.” Each item was rated on a 7-point scale (1 = *strongly disagree*, 7 = *strongly agree*) with item 4 reverse scored (see Form A1 in Supplementary Information). Scores range from 6 to 42 with higher scores indicating higher levels of satisfaction. An exploratory factor analysis revealed one large factor that explained 67.51% of the total variance and the factor loadings of the six items ranged between 0.79 and 0.85, suggesting that each item substantially contributes to the factor at fair and excellent levels. Internal consistency of the GMSQ in the present sample was excellent (Cronbach’s *α* = 0.90).

#### COVID-19 Risk Perception Questionnaire

The perceived risk of COVID-19 during the outbreak was measured by the Risk Perception Questionnaire (RPQ) adapted from Oh and colleagues^56^ by replacing the word “Middle East Respiratory Syndrome coronavirus (MERS-CoV)” with “COVID-19” (see Form A2 in Supplementary Information). The PRQ consisted of 4 items: (1) I consider COVID-19 to be a serious problem; (2) I am worried that I will be affected by COVID-19; (3) It is likely that I will be affected by COVID-19; and (4) I feel that COVID-19 is dangerous. Each item was rated on a 7-point scale (1 = *strongly disagree*, 7 = *strongly agree*). Scores range from 4 to 28 with higher scores indicating greater personal levels of risk perception. Internal consistency was excellent for the original RPQ (Cronbach’s *α* = 0.92)^56^, and almost acceptable for the adapted RPQ in the present sample (Cronbach’s *α* = 0.69).

#### Gratitude Questionnaire

The Gratitude Questionnaire (GQ) developed by McCullough and colleagues^57^ was used to assess the disposition toward gratitude: “(1) I have so much in life to be thankful for; (2) If I had to list everything that I felt grateful for, it would be a very long list; (3) When I look at the world, I don’t see much to be grateful for; (4) I am grateful to a wide variety of people; (5) As I get older, I find myself more able to appreciate people, events, and situations that have been part of my life history; and (6) A significant amount of time can pass before I feel grateful for something or someone.” Each item was rated on a 7-point scale (1 = *strongly disagree*, 7 = *strongly agree*) with items 3 and 6 reverse scored (see Form A3 in Supplementary Information). Scores range from 6 to 42 with higher scores indicating higher levels of gratitude. It has been proved that the GQ has good psychometric properties with high reliability and validity in Chinese populations^58^.

#### S-Anxiety subscale of State-Trait Anxiety Inventory

The experiences and feelings of fear, stress, apprehension, and neuroticism (i.e. state anxiety) during the pandemic were measured by State-Anxiety subscale of The State Anxiety Inventory (STAI-S) developed by Spielberger^59^. Sample items include: “I feel frightened; I am worried.” The STAI-S consists of 20 items and is scored on a 4-point scale (1 = *not at all,* 4 = *very significant*) with items 1, 2, 5, 8, 10, 11, 15, 16, 19, and 20 reverse scored (see Form A4 in Supplementary Information). Scores range from 20 to 80 with higher scores indicating greater levels of state anxiety. The STAI possesses acceptable psychometric properties to measure anxiety in Chinese culture^60^.

### Statistical analyses

All data in the present study were processed using SPSS version 25.0 (IBM Corp, Armonk, NY, USA). After descriptive statistics, Pearson correlation tests with Bonferroni correction (× 6) were performed to evaluate the association between GMSQ, RPQ, GQ, and STAI-S.

Next, Direction Dependence Analysis (DDA) was applied to evaluate the most probable causal direction between variables using the SPSS add-ons (publicly available at https://www.ddaproject.com). The DDA was developed to determine causal relationships^61–63^ and has been used effectively in previous studies^64–66^. Specifically, the DDA consists of three components and the target model (e.g. x → y) finds support when (1) the distribution of dependent variable y is closer to normality than the distribution of independent variable x, (2) the residual distribution of target model (x → y) is closer to normality than the residuals of causally reversed model (e.g. y → x), and (3) the independence assumption of residuals and predictors holds for target model is violated for the reversed model. In the current study, three hypothesized target models (GMSQ → RPQ, GMSQ → GQ, and GMSQ → STAI-S) and corresponding reversed models (RPQ → GMSQ, GQ → GMSQ, and STAI-S → GMSQ) were tested by DDA model selection while controlling for basic demographic variables (gender, age, current residency, education attainment) as covariates.

Multiple parallel mediation modeling was performed using the PROCESS macro v3.3 for SPSS (Model 4)^67^ to explore whether satisfaction with government management (GMSQ) predicted public anxiety (STAI-S) and whether risk perception (RPQ) and gratitude (GQ) mediated this relationship. Basic demographic variables (gender, age, current residency, education attainment) were all inputted as covariates for the analysis. The bootstrap method was applied with 5000 resamples and bias-corrected 95% confidence intervals (CI). Confidence intervals without zero indicate significant mediating effects.

## Results

### Descriptive statistics and correlation analysis

The average anxiety level assessed from March 25 to 30, 2020 in the present study (*M* = 37.38, *SD* = 11.91) was decreased as compared to that assessed during the peak period (Jan 24 to Feb 24, 2020) in the previous study (*M* = 48.7, *SD* = 10.8)^68^. Participants were highly satisfied with government management of the pandemic (*M* = 39.92, *SD* = 3.80; possible scores range from 6 to 42), and reported high levels of gratitude (*M* = 32.32, *SD* = 5.51; possible scores range from 6 to 42) and risk perception of illness (*M* = 21.60, *SD* = 4.55; possible scores range from 4 to 28).

State anxiety level was significantly positively associated with risk perception of COVID-19 (*r* = 0.25, *p* < 0.001) but negatively associated with the disposition toward gratitude (*r* = −0.34, *p* < 0.001) and management satisfaction (*r* = −0.18, *p* < 0.001). Meanwhile, management satisfaction was significantly positively correlated with risk perception of COVID-19 (*r* = 0.21, *p* < 0.001) and the disposition toward gratitude (*r* = 0.28, *p* < 0.001). No significant correlation was found between the disposition toward gratitude and risk perception of COVID-19 (*r* = 0.04, *p* = 0.20). Table 3 showed the means and standard deviations for each questionnaire, as well as Pearson correlation coefficients among these variables.

**Table 3:** Descriptive statistics and correlation analysis for questionnaires (N = 808).

Variable	*M*	*SD*	1	2	3	4
Management satisfaction	39.92	3.80	–			
Risk perception of COVID-19	21.60	4.55	0.21**	–		
Disposition toward gratitude	32.32	5.51	0.28**	0.04	–	
State anxiety	37.38	11.91	−0.18**	−0.25**	0.34**	–

**p < 0.01 Bonferroni-corrected (× 6).

### Direction dependence analysis

DDA results indicated that the causal models where management satisfaction as the predictor while risk perception of COVID-19, the disposition toward gratitude, and state anxiety level as outcomes (i.e., GMSQ → RPQ, GMSQ → GQ, and GMSQ → STAI-S) were more likely to approximate the underlying data-generating mechanism than the causally reversed models when controlling for basic demographic covariates. Detailed DDA results were given in Table 4. First, results for observed variable distributions showed significant differences in skewness and kurtosis for all three DDA model selections, where the GMSQ variable was more skewed and heavy-tailed than RPQ, GQ, and STAI-S variables, which suggested GMSQ should be the independent variable. Second, results for distributional characteristics of model residuals also showed significant differences in skewness and kurtosis for all three model selections, where residuals of corresponding reversed models were more skewed and heavy-tailed than target models, which pointed in the same causal directions. Last, the independence assumption of residuals and predictors was examined by the Breusch-Pagan homoscedasticity test (BP test). A non-significant p-value indicates the model is more likely to have the ‘true’ direction of effect when its reversed model shows a significant p-value. Results of BP tests supported the GMSQ → STAI-S and GMSQ → RPQ directions as indicated by non-significant target models and significant reversed models. With regard to DDA model selections for GMSQ → GQ, although the BP test rejected the independence assumption in both target model and reversed model, DDA indicators for observed variable and residual distributions did provide at least partial support for the GMSQ → GQ direction.

**Table 4:** Results of direction dependence analysis.

DDA properties	Target models		
	GMSQ → STAI-S	GMSQ → RPQ	GMSQ → GQ
Variable distributions			
Skewness diff (95% CI)^a^ Kurtosis diff (95% CI)^a^	2.77 [1.82, 3.55] 15.70 [6.21, 24.48]	2.59 [1.63, 3.35] 15.52 [5.23, 23.17]	2.79 [1.83, 3.54] 15.94 [5.70, 23.44]
Residuals distributions			
Skewness diff (95% CI) ^a^ Kurtosis diff (95% CI) ^a^	2.74 [1.61, 3.67] 16.68 [5.37, 26.40]	2.36 [1.41, 3.09] 14.03 [4.76, 21.35]	2.89 [1.57, 4.01] 17.33 [5.04, 27.11]
Independence			
P_h_ in target model^b^ P_h_ in reversed model^b^	0.364 < 0.001	0.376 < 0.001	< 0.001 < 0.001
DDA decision	Target model	Target model	Target model (weak)

Demographic variables (gender, age, current residency, education attainment) were all inputted as covariates for direction dependence analysis (DDA).

*GMSQ* the self-developed Government Management Satisfaction Questionnaire scores, *STAI-S* State-Anxiety subscale scores of the State Anxiety Inventory, *RPQ* the Risk Perception Questionnaire scores, *GQ* the Gratitude Questionnaire scores.

^a^Nonparametric bootstrap approach based on 1000 bootstrap resamples and 95% confidence interval (CI).

^b^P_h_ is p value for Breusch–Pagan heteroscedasticity test.

### Mediation model analysis

**Figure 2:**
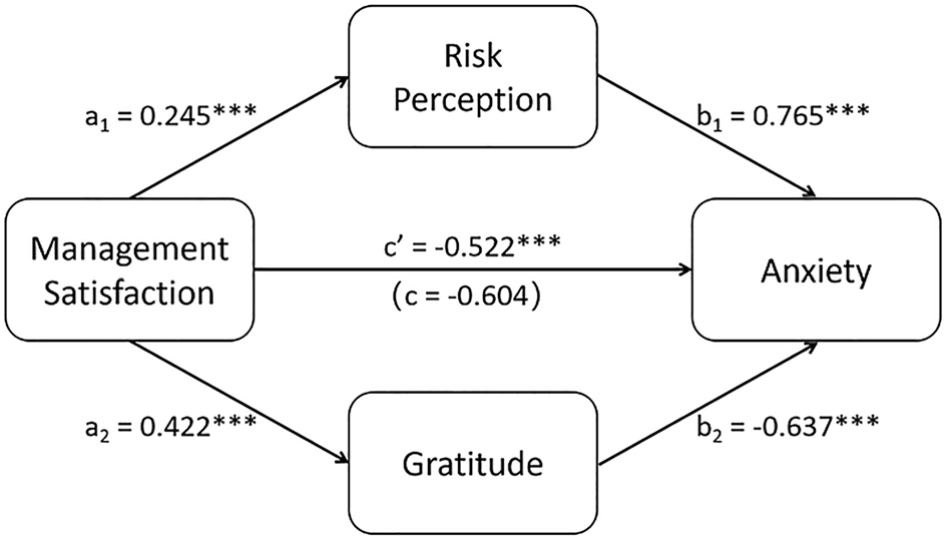
Parallel-multiple mediation of gratitude and risk perception between management satisfaction and anxiety. ***p < 0.001.

Figure 2 presented the findings for the parallel mediating roles of gratitude and risk perception in the relationship between management satisfaction and public anxiety. Overall, findings indicated that management satisfaction could significantly directly predict public anxiety (direct effect: *c*^*’*^ = −0.522, *p* < 0.001), meanwhile the relationship between management satisfaction and public anxiety was partially mediated by risk perception and gratitude.

People who showed higher satisfaction with government control actions were more likely to have higher risk perception of COVID-19 (a_1_ = 0.245, *t*(802) = 5.919, *p* < 0.001), and increased risk perception was related to higher levels of public anxiety (b_1_ = 0.765, *t*(800) = 9.043, *p* < 0.001). The indirect path of management satisfaction on public anxiety through risk perception was significantly positive (a_1_b_1_ = 0.187, 95% CI = [0.116, 0.272]).

On the other hand, people who showed higher satisfaction with government control actions were more likely to possess greater levels of gratitude (a_2_ = 0.422, *t*(802) = 8.599, *p* < 0.001), and more gratitude was related to less anxiety (b_2_ = -0.637, *t*(800) = 8.942, *p* < 0.001). The indirect path of management satisfaction on public anxiety through gratitude was significantly negative (a_2_b_2_ = −0.269, 95% CI = [−0.386, −0.167]).

## Discussion

The reduction of anxiety right now is more important than ever while the world is fighting COVID-19. The current study was performed during the period when newly confirmed cases in China had significantly declined^49–51^. This period is timely in capturing how positive attitudes toward government management affect public anxiety, risk perception, and gratitude. Our findings maintain that satisfaction with the government is directly related to lower levels of public anxiety. The strength of this relationship might be indirectly reduced by risk perception and indirectly increased by gratitude. These results may inspire potential strategies for governments to reduce anxiety for the public during the pandemic.

The results of the current anonymous and confidential online survey showed high levels of satisfaction with the Chinese government’s management of the COVID-19 pandemic. Compared with the SARS crisis in 2002, more rapid and robust responses were taken by the government during the COVID-19 pandemic, such as better prepared public health systems, earlier announcements of public health emergencies, and more aggressive quarantine measures^69,70^, which were proved to curb the spread effectively^49–51^. As a report by World Health Organization (WHO) notes, “this rather unique and unprecedented public health response in China reversed the escalating cases”^71^. China’s response not only exceeds public expectations but also provides important lessons for global response^72–74^. Thus, high levels of public satisfaction with government management may result from the public’s perceptions of performance far exceeding their expectations. Consistently, Wu and his colleagues also reported high levels of satisfaction with government performance during COVID-19 in China^21^. And they further indicated that public satisfaction was not only impacted by actual government performance, but also by authoritarian control and political culture^21^. Previous studies on institutional trust also reported significant associations with authoritarianism and social dominance orientation^22,75^. Therefore, it should be further verified whether political reasons contribute part of high levels of satisfaction.

The present study reported reduced anxiety as compared to that assessed during the peak period (Jan 24 to Feb 24, 2020)^68^ and satisfaction with government management could directly negatively predict public anxiety^23^. It is in line with most studies on life satisfaction^24,76^, as well as a recent study on social workers during the pandemic^23^. As stated by the uncertainty and anticipation model^77^, “Anxiety is characterized by anticipatory cognitive, behavioral and affective changes in response to uncertainty about potential threat”. Due to the uncontrollability, invisibility and fatality of COVID-19, public anxiety was higher during the peak period^68^. And with the rapid and robust responses taken by the government, the spread slowed down^49–51^. Higher satisfaction with government control actions during this period indicated less uncertainty caused by COVID-19 and thereby reduced anxiety. The current finding emphasizes the importance of improving satisfaction with the government during the pandemic for mental health.

The majority of people’s perceived risk in the current study reached high levels. Previous studies reported lower risk perception of infection^5,78^, but consistently higher risk perception during the earlier period of the current survey (February to March 2020) in China^29,79^. It is also in line with the study in national samples across ten countries during the same period of the current survey (March to April 2020)^80^. In the present study, risk perception partially positively mediated the effect of management satisfaction on anxiety. Individuals who showed higher satisfaction with government control actions would be more likely to follow the policies and take protective actions, such as mask-wearing, handwashing, and social distancing. When individuals are more engaged in prevention, they will get more knowledge of the virus and their affective experiential system is dominating in processing risk perceptions, thereby increase their risk perceptions and foster public anxiety^9,81,82^.

The level of gratitude in the current study is high, which is consistent with the finding that gratitude is a common positive psychological process in post-disaster situations^37^. To our knowledge, the present study is the first to investigate the state of gratitude among the general public of China during the crisis period. And the finding that gratitude plays a negative partial mediation role between satisfaction with government management and anxiety not only extends the association between life satisfaction and gratitude^47,48^ but also has the potential clinical implication during the COVID-19 pandemic. Substantial empirical research reported the effectiveness of gratitude interventions in reducing anxiety symptoms^42,44–46^. Gratitude and mindfulness are related abilities^83,84^, and indeed a recent study did show that mindfulness-based stress reduction protocol can support psychological well-being during the COVID-19 lockdown^85^.

There are several limitations to this study. First, this study employed a cross-sectional design that could not establish strong causal relationships^86^. Although the DDA approach was applied to evaluate the most probable causal direction in the current study, further laboratory or longitudinal studies are needed to demonstrate strong direct causal relationships considering the opposite direction was also reported in previous cross-sectional studies (e.g. high perceived anxiety during hazards enhanced risk perceptions^87,88^). Second, this study adopted a convenience sampling approach to collect the data, which may not be representative of the entire Chinese population. Third, the web-based survey may be influenced by social desirability bias, especially by those who are familiar with response forms. Finally, the Government Management Satisfaction Questionnaire and COVID-19 Risk Perception Questionnaire developed in this study were used for the first time as no other validated measures were available. Future research requires a comprehensive evaluation to determine the reliability and validity of the scales.

## Conclusion

This study showed that satisfaction with government management may reduce public anxiety, with risk perception and gratitude playing a partially mediating role. The findings have several implications for policymakers to implement intervention programs to protect the public’s mental health and facilitate the management of the COVID-19 pandemic. Firstly, the authorities could reduce public anxiety by enhancing public satisfaction, such as reducing expectations of government performance and increasing the feeling of trust toward the government. Second, although high levels of risk perception are associated with adopting precautionary actions, this may also elicit anxiety, PTSD^89^, and depression^29^. When the perceived threat of the pandemic far outweighs the real danger, the government needs to adjust the public’s risk perception to avoid excessively stressful behaviors and emotions. Finally, management satisfaction may also result in a rise in gratitude to promote the reduction of anxiety. Our results high lights the importance role of positive cognition and emotion in mitigating anxiety during the pandemic (Supplementary Information).

## Supplementary Information

Supplementary Information.

## Data Availability

Derived data supporting the findings of this study are available from the corresponding author Y. L on request.
