# Supplementary material for: Risk perception and gratitude mediate the negative relationship between COVID-19 management satisfaction and public anxiety
Source: Sci Rep. 2023 Feb 27;13:3335. doi: 10.1038/s41598-023-29815-2 (PMC9969377; doi:10.1038/s41598-023-29815-2)
Supplement: Supplementary file 1 — Supplementary Information. [file 41598_2023_29815_MOESM1_ESM.docx]

Appendix

Form A1

*Government Management Satisfaction Questionnaire*

Introduction: Please read the following items carefully and choose the number which accurately describes your current feelings.

1 = strongly inconsistent

2 = consistent

3 = slightly consistent

4 = unsure

5 = slightly consistent

6 = consistent

7 = strongly consistent

1. The current pandemic would be awful without government actions;

(2) I am very appreciative of what the state has done to prevent and control the pandemic;

(3) I am proud of our current pandemic prevention and control achievements;

(4) I think the pandemic continues primarily because the government’s prevention and controls are inadequate;

(5) I support the government’s pandemic prevention and control actions;

(6) To prevent and control the pandemic, I am positively cooperating with the government.

Form A2

*COVID-19 Risk Perception Questionnaire*

Introduction: Please read the following items carefully and choose the number which accurately describes your current feelings.

1 = strongly disagree

2 = disagree

3 = slightly disagree

4 = neutral

5 = slightly agree

6 = agree

7 = strongly agree

(1) The problem of COVID-19 is serious to me;

(2) I am worried that I could be affected by COVID-19;

(3) It is likely that I will be affected by COVID-19;

(4) I have felt that COVID-19 is dangerous.

Form A3

*Gratitude Questionnaire*

Introduction: Please read the following items carefully and choose the number which accurately describes your current feelings.

1 = strongly disagree

2 = disagree

3 = slightly disagree

4 = neutral

5 = slightly agree

6 = agree

7 = strongly agree

(1) I have so much in life to be thankful for;

(2) If I had to list everything that I felt grateful for, it would be a very long list;

(3) When I look at the world, I don’t see much to be grateful for;

(4) I am grateful to a wide variety of people;

(5) As I get older, I find myself more able to appreciate people, events, and situations that have been part of my life history;

(6) A long time can pass before I feel grateful for something or someone.

Form A4

*S-Anxiety subscale of State-Trait Anxiety Inventory*

Introduction: Please read the following items carefully and choose the number which accurately describes your current feelings.

1 = Not at all

2 = A little

3 = Somewhat

4 = Very much so

1. I feel calm;
2. I feel secure;
3. I feel tense;
4. I feel strained;
5. I feel at ease;
6. I feel upset;
7. I am presently worrying over possible misfortunes;
8. I feel satisfied;
9. I feel frightened;
10. I feel uncomfortable;
11. I feel self-confident;
12. I feel nervous;
13. I feel jittery;
14. I feel indecisive;
15. I am relaxed;
16. I feel content;
17. I am worried;
18. I feel confused;
19. I feel steady;
20. I feel pleasant;
